# Kinetics of Pectin Biopolymer Facial Erosion Characterized by Fluorescent Tracer Microfluidics

**DOI:** 10.3390/polym14183911

**Published:** 2022-09-19

**Authors:** Matthew W. Liao, Betty S. Liu, Joseph Sutlive, Willi L. Wagner, Hassan A. Khalil, Zi Chen, Maximilian Ackermann, Steven J. Mentzer

**Affiliations:** 1 Laboratory of Adaptive and Regenerative Biology, Brigham & Women’s Hospital, Harvard Medical School, Boston, MA 02115, USA; 2 Translational Lung Research Center, Department of Diagnostic and Interventional Radiology, University of Heidelberg, 69117 Heidelberg, Germany; 3 Institute of Functional and Clinical Anatomy, University Medical Center of the Johannes Gutenberg-University Mainz, 55131 Mainz, Germany

**Keywords:** pectin, tannic acid, kinetics, biostability, biodegradation, erosion, microfluidics, microfluorimetry, cohesion, adhesion

## Abstract

Pectin is a plant-derived heteropolysaccharide that has been implicated in drug development, tissue engineering, and visceral organ repair. Pectin demonstrates remarkable biostability in a variety of physiologic environments but is biodegradable in water. To understand the dynamics of pectin biodegradation in basic environments, we developed a microfluidics system that facilitated the quantitative comparison of pectin films exposed to facial erosion. Pectin biodegradation was assessed using fluorescein tracer embedded in pectin, trypan blue quenching of released fluorescence, and highly sensitive microfluorimetry. The microfluidic perfusate, delivered through 6 um-pore synthetic membrane interface, demonstrated nonlinear erosion of the pectin film; 75% of tracer was released in 28 h. The microfluidics system was used to identify potential modifiers of pectin erosion. The polyphenolic compound tannic acid, loaded into citrus pectin films, demonstrated a dose-dependent decrease in pectin erosion. Tannic acid had no detectable impact on the physical properties of citrus pectin including adhesivity and cohesion. In contrast, tannic acid weakened the burst strength and cohesion of pectins derived from soy bean and potato sources. We conclude that facial erosion may explain the biostability of citrus pectin on visceral organ surfaces as well as provide a useful method for identifying modifiers of citrus pectin biodegradation.

## 1. Introduction

Pectin is a biopolymer that has been implicated in drug development, tissue engineering, and visceral organ repair [1,2,3]. A plant-derived heteropolysaccharide, pectin is the most abundant polysaccharide in the plant cell wall and middle lamella. Pectin’s branched-chain structure forms a strong but extensible matrix that accommodates the effect of turgor and contributes to the physical characteristics of plants [4,5]. Chemically, pectins are the most complex cell wall glycans [6]. Pectins are composed of partial methyl esters of (1–4)-alpha-d-galacturonic acid interrupted with rhamnose units and other neutral sugars [6].

Relevant to biomedical applications, pectin demonstrates distinctive physicochemical properties in varied physiologic environments. For example, pectin has the ability to form gels in acid environments [7]. Acid resistance allows pectin to maintain its structure in the gastric lumen. Pectin is also resistant to proteases and amylases [8]. Enzyme resistance allows pectin to maintain its structural integrity in foregut and duodenal secretions. In contrast to these examples of biodurability, pectin is biodegradable in a basic environment. Pectin films rapidly dissolve when immersed in water.

A physiologic environment that is not well-understood is the lung’s pleural surface. High methoxyl citrus pectin has been shown to intimately bind to the pleural glycocalyx [9,10]. Pectin’s adhesivity and extensibility are valuable properties in a lung sealant [11]. Pectin effectively seals pleural injuries while accommodating breathing-related variation in lung volume and surface areas [3,12]. Although normal pleural fluid provides a basic environment [13], pectin biodurability half-life is estimated at 7 days [12]. These findings indicate that pectin’s pleural fluid exposure is not equivalent to immersion testing. The durability of the pectin-glycocalyceal interaction suggests that pectin bound to the lung surface has limited erosion exposure.

To explore the dynamics of pectin biodegradation in a setting of facial exposure, we developed a microfluidics system that permitted multiple parallel comparisons of pectin films exposed to facial erosion; that is, exposure limited to a water interface. Distinct from immersion testing, facial erosion facilitated the characterization of tannic acid as a modifier of pectin biodegradation.

## 2. Materials and Methods

### 2.1. Pectin

The citrus pectins used in this study were obtained from a commercial source (Cargill, Minneapolis, MN, USA). The citrus pectins were high-methoxyl pectins (HMP); that is, pectin polymer with greater than 50% degree of methoxylation. Soy bean and potato pectins were obtained from Megazyme (Bray, Ireland). The pectin powder was stored in low humidity at 25 °C prior to use.

### 2.2. Pectin Dissolution in Water

The pectin powder was dissolved by a step-wise increase in added water to avoid undissolved pectin [14]. Swelling and softening of the particles were followed by fluidization and dissolution [15]. The complete dissolution of the pectin was achieved by a high-shear 10,000 rpm rotor-stator mixer (L5M-A, Silverson, East Longmeadow, MA, USA). To ensure reproducible mixing, viscosity monitoring was performed with a digital tachometer and ammeter (DataLogger, Silverson). The dissolved pectin was poured into a standard mold for further studies.

### 2.3. Tracer Embedding

The fluorescent tracer fluorescein sodium (C_20_H_10_Na_2_O_3_; MW 376.27), obtained in powder form (Sigma-Aldrich, St. Louis, MO, USA), was dissolved in water to create a 1 mg/mL concentrated stock solution of fluorescein. The fluorescein tracer was loaded into the pectin during high-shear mixing.

### 2.4. Fluorescent Measurement

The fluorescein tracer embedded in the pectin film was measured with a CytoFluor 4000 Fluorescence Measurement System (Millipore, Bedford, MA, USA). The CytoFluor 4000 is a computer-controlled multi-well fluorescence scanning device with a tungsten halogen lamp and a broadband interference filter for fluorescein: excitation 450 ± 50 nm and emission 530 ± 25 nm. Intensity readings were obtained from the bottom of the microfluidics plate as relative fluorescence unites (RFU). Background fluorescence (medium alone) was measured separately for each plate and subtracted from all readings. The data were exported to the Microsoft Excel 360 (Redmond, WA, USA) spreadsheet for data analysis. Each experiment was performed in triplicate or quadruplicate. The standard deviation (SD) of replicate measures was uniformly less than 30% of the mean.

### 2.5. Quenching Agent

Trypan blue solution (C_34_H_24_N_6_Na_4_O_14_S_4_; MW 960) was obtained in a 0.4% solution in 0.81% NaCl and 0.06% KPO_4_ (Sigma). The trypan blue was used at a final concentration of 0.04% (*w*/*v*) or as indicated.

### 2.6. Tannic Acid

The polyphenolic compound tannic acid (C_76_H_52_O_46_; MW 1701.20) was obtained from commercial sources as an undissolved powder (Sigma-Aldrich, St. Louis, MO, USA). The tannic acid was loaded into the citrus pectin at concentrations of 0%, 0.27%, 0.81%, 1.35%, 1.62%, and 1.89% *w*/*w* in water, using a high-shear mixer.

### 2.7. Microperfusion

The microperfusion system was controlled with New Era Pump Systems NE-1800 multi-channel programmable syringe pump (Farmingdale, NY, USA). In most experiments, 1 mL syringes (BH Supplies, Jackson, NJ, USA) loaded with 0.4% trypan blue dye perfused the matrix interface through 0.46 mm-diameter polyethylene tubing (Instech, Plymouth Meeting, PA, USA) at 1 uL/h. The circular 14 mm-diameter tracer embedded pectin films were placed in the bottom of a 24-multiwell clear flat bottom cell culture plate (Falcon, Corning, NY, USA) and overlaid with a circular (12 mm-diameter) black 6 um-pore matrix (Thomas Scientific, Swedesboro, NJ, USA) loaded with 17 uL of 0.4% trypan blue prior to constant rate perfusion.

### 2.8. Adhesion Testing

Adhesion testing was performed as previously described [11]. Briefly, polymer-polymer adhesion experiments were performed with a custom fixture designed for the TA-XT plus with a 50 kg load cell (Stable Micro Systems, 110 Godalming, Surrey, UK). The fixture was composed of a 25 mm-diameter flat-ended acrylic cylindrical probe and a flat acrylic fixture surface. The 20 mm-diameter films, affixed to both surfaces, were hydrated with 7 uL of distilled water. The cylindrical probe compressed the polymers at a force of 5 N and a development time of 10 s followed by probe withdrawal at 0.2 mm/s. Data was acquired at a rate of 500 points per second (pps).

### 2.9. Cohesion Testing

Fracture mechanics were performed as previously described [11]. Briefly, the biopolymers were subjected to a controlled uniaxial load normal to the plane of the polymer film. The load was applied with a 5 mm stainless-steel spherical probe mounted to a TA-XT plus (Stable Micro Systems) with a 50 kg load cell. The stainless-steel ball was positioned centrally over the biopolymer. The probe compressed the biopolymers at a test speed of 2 mm/s until fracture. The fracture force, distance, and time were recorded at 500 pps.

## 3. Results

### 3.1. Fluorescein Tracer

**Figure 1:**
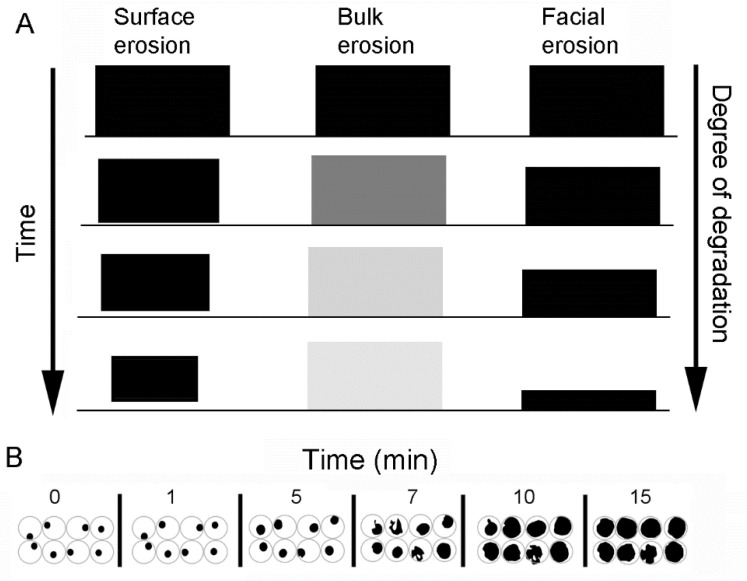
Schematic illustration of the different modes of mass loss from degradable polymers. (**A**) Conventional understanding of polymer degradation includes surface erosion and bulk erosion [16]. Facial erosion describes the degradation of polymers adherent to visceral organ surfaces. (**B**) Example of circular pectin films immersed in water. The pectin films demonstrate progressive swelling and dissolution.

Biodegradation decreases polymer mass as a function of time. In addition to the commonly recognized mechanisms of surface and bulk erosion, there are selected physiologic environments that reflect facial erosion (Figure 1A). A common method for evaluating surface and bulk erosion is water immersion. When pectin film biodegradation was assessed by water immersion, there was a rapid swelling and dissolution of the films (Figure 1B). The rapidity of degradation precluded effective analysis of the process.

**Figure 2:**
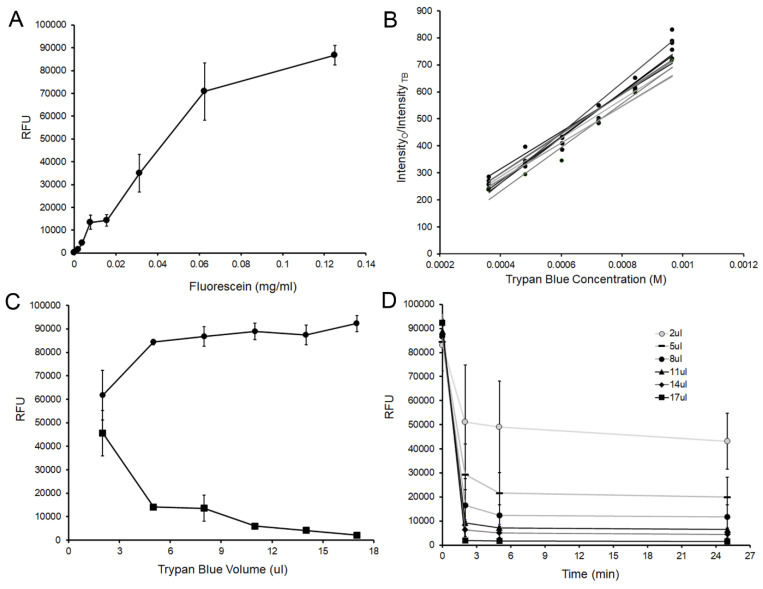
Characterizing the fluorescein fluorescence and trypan blue quenching. (**A**) The fluorescein standard curve was linear at lower concentrations. Modest nonlinearity at the higher concentrations was likely due to auto-quenching and detector saturation. (**B**) Trypan blue was used to quench the released fluorescein tracer. The effectiveness of trypan blue quenching was assessed over a 2-fold range of fluorescein concentrations from 1.95 ug/mL (light gray line) to 125 ug/mL (black line). At these fluorescein concentrations, the experimentally obtained Stern-Vollmer plot was linear with an aggregate constant of 7.15 × 10^5^ M^−1^ (R^2^ = 0.9754). (**C**) Using standard 0.4% trypan blue solutions, the quenching of 125 ug/mL of fluorescein (circles) was assessed at increasing volumes of trypan blue (squares). Greater than 90% of the detectable fluorescence was reduced above 5 uL of trypan blue. (**D**) The trypan blue-associated quenching was rapid and stable. Maximal quenching occurred within seconds of the addition of the trypan blue at all volumes from 2 uL (light gray line) to 17 uL (black line). Error bars = 1 SD.

To quantitatively assess facial erosion of pectin films, we evaluated fluorochromes for their potential utility as tracers for pectin biodegradation. Fluorescein was readily detected by microfluorimetry and was linear over a wide concentration range (Figure 2A). The fluorescein molecules demonstrated no evidence of biochemical reactivity with pectin films and were efficiently quenched with trypan blue (Stern-Vollmer constant 7.15 × 10^5^ M^−1^) (Figure 2B). Using standard 0.4% trypan blue solutions, 4 uL of a standard concentration of fluorescein (125 ug/mL) was effectively quenched by 5 uL of trypan blue (Figure 2C). The quenching was stable over time (Figure 2D).

### 3.2. Free Volume Embedding

**Figure 3:**
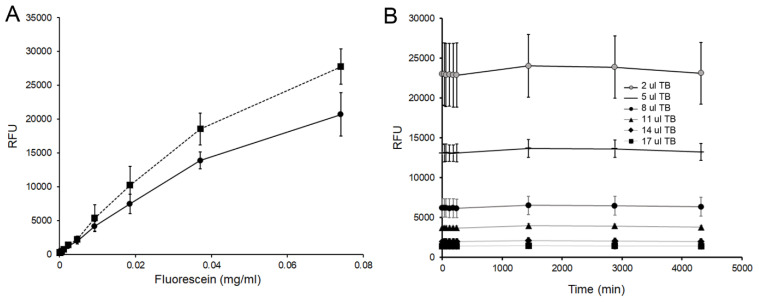
Fluorescein tracer embedded into the pectin films. (**A**) Pectin films embedded with fluorescein demonstrated a mostly linear relationship to fluorescein concentration (solid line). A slight increase in fluorescence was noted after pectin dissolution with distilled water (10 uL) (dotted line). (**B**) Tracer fluorescence, at a variety of concentrations, was stable for 72 h. Serial dilutions are shown from 37 ug/mL (black line) to 2.3 ug/mL (light gray line). Error bars = 1 SD.

To determine the effect of pectin embedding on the fluorescein signal, increasing concentrations of fluorescein were embedded into the pectin films. Fluorimetry demonstrated a largely linear increase over a significant range of concentrations (Figure 3A). Solubilization of the pectin film produced a modest, but proportional, increase in fluorescence intensity–a change likely reflecting concentration-dependent auto-quenching (Figure 3A). A range of fluorescein concentrations were stable in the embedded films for more than 72 h (Figure 3B).

### 3.3. Matrix Interface

**Figure 4:**
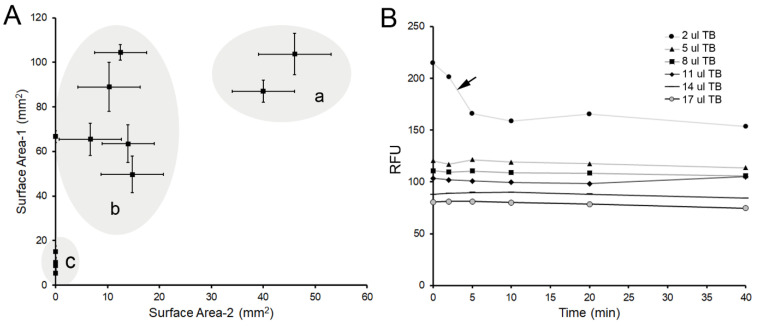
A variety of laboratory materials were empirically evaluated for matrix characteristics suitable for evaluating facial erosion. (**A**) We screened a variety of commercial and laboratory porous matrices typically used as filters. Double-thickness matrices were used to evaluate lateral and penetrating diffusion of the trypan blue perfusate. The surface area of the superficial matrix (Surface Area-1) was compared to the surface area of the deeper layer (Surface Area-2). Commercial filters with pore sizes between 40–50 um resulted in the greatest surface and penetrating diffusion (a). Laboratory filters, generally composed of nitrocellulose and cellulose acetate, had pore sizes between 6 and 10 um. These filters had comparable surface diffusion, but significantly less penetrating diffusion (b) than the larger pore matrices (a). Specialty filters, composed of synthetic microfibers, nylon membranes, and biologic matrices (e.g., collagen), demonstrated limited trypan blue diffusion (c). (**B**) After initial application of the trypan blue, the optical properties of the matrices were stable over 40 min. A notable exception was the fluorescence measured at low concentrations of trypan blue (arrow). Because of the potential reflectance artifact from the white matrices, a black 112 mm^2^ × 160 um matrix with 6 um-pore size was used for subsequent experiments (CAT#: 4740C10, Thomas Scientific). Error bars = 1 SD.

To obtain a wettable and erosive interface, we screened a variety of porous matrices for their ability to disperse the microfluidic perfusate. Most of the matrices were cellulose-based (cellulose acetate and nitrocellulose) filters, but a variety of nylon and synthetic membranes were screened as well (Figure 4A). To evaluate lateral and penetrating diffusion, double-thickness matrices were tested. The surface area of the superficial matrix (Surface Area -1) was compared to the surface area of the deeper layer (Surface Area-2). The ideal matrix maximized lateral diffusion while minimizing penetrating diffusion. The matrices with both lateral diffusion and penetration (Figure 4A (a)) provided a less well-developed interface when compared to synthetic membranes (Figure 4A (b)) or cellulose-based filters (Figure 4A (c)). Synthetic 6 um-pore membranes were found to provide an optimal interface. To minimize light reflectance artifact, a black membrane was used.

### 3.4. Microfluidics of Facial Erosion

**Figure 5:**
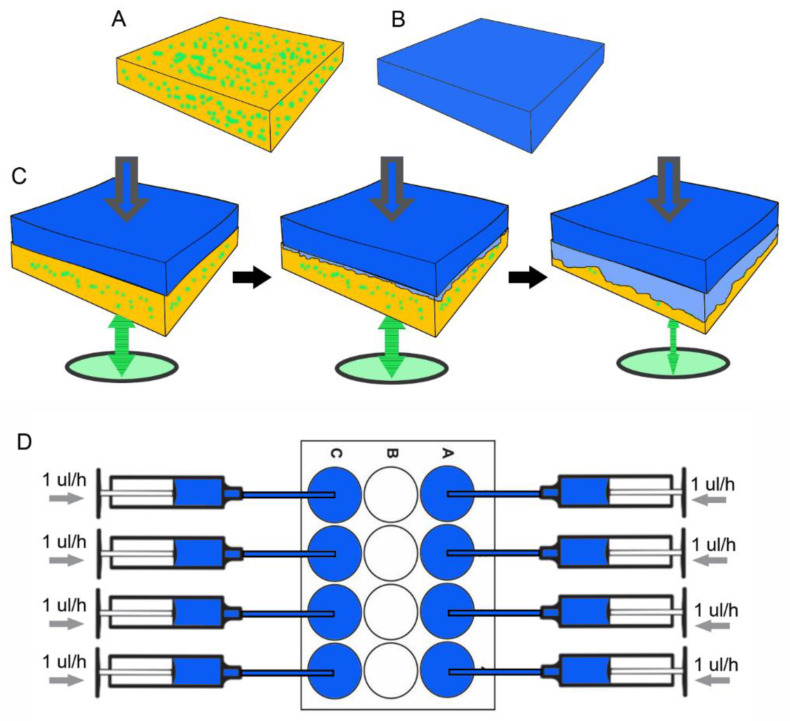
Schematic of facial erosion assay. (**A**) Pectin with fluorescein tracer embedded in pectin free volume. (**B**) Trypan blue loaded into cellulose-based 6 um-pore filter matrix (12 mm diameter) providing the interface. (**C**) The contact of the trypan blue matrix and the fluorescein-labeled pectin results in gradual loss of fluorescein from the pectin matrix. Loss of the fluorescein is quantitatively assessed by the microfluorimeter from the bottom of the plate (green arrow). (**D**) A microfluidics syringe pump perfusion system allowed for the computer-controlled parallel perfusion of 8 samples. Most samples were perfused at 1 uL/h.

**Figure 6:**
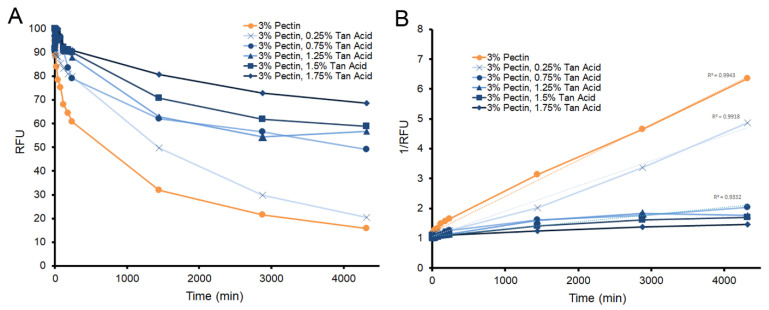
Dose-dependent effect of tannic acid on pectin film degradation over 3 days of continuous microperfusion. (**A**) Substantial pectin facial erosion occurs over the first day (orange). (**B**) Surface erosion demonstrated reproducible kinetics. Plots of 1/RFU demonstrated straight lines for all samples (R^2^ ranged from 0.9943 to 0.9332). Facial erosion half-life for pectin alone was estimated at 14 h. The estimated half-life of pectin increased with increasing tannic acid concentration: 0.25%, 20 h; 0.75%, 69 h; 1.25%, 79 h; 1.5%, 99 h; and 1.75%, 154 h.

The microfluidics system consisted of fluorescein-embedded pectin polymers (Figure 5A) overlaid with a black porous matrix (Figure 5B) perfused with 0.4% trypan blue (Figure 5C). Serial microfluorimetry measurements were recorded from the pectin aspect of the interface. The system used 8 parallel syringe pumps perfusing the interface at 1 uL/h (Figure 5D). When citrus pectin films were studied, there was nonlinear erosion of the pectin film (Figure 6A, orange); the tracer was reduced by 75% in 28 h. To demonstrate the potential effect of a crosslinker on pectin erosion, the molecule tannic acid was added to the film. Tannic acid is an important member of the natural polyphenol family [17,18]. When added to pectin films, tannic acid demonstrated a dose-dependent decrease in pectin erosion. When plotted as the inverse of residual fluorescence (1/RFU), the pectin tracer demonstrated linear kinetics (Figure 6B).

**Figure 7:**
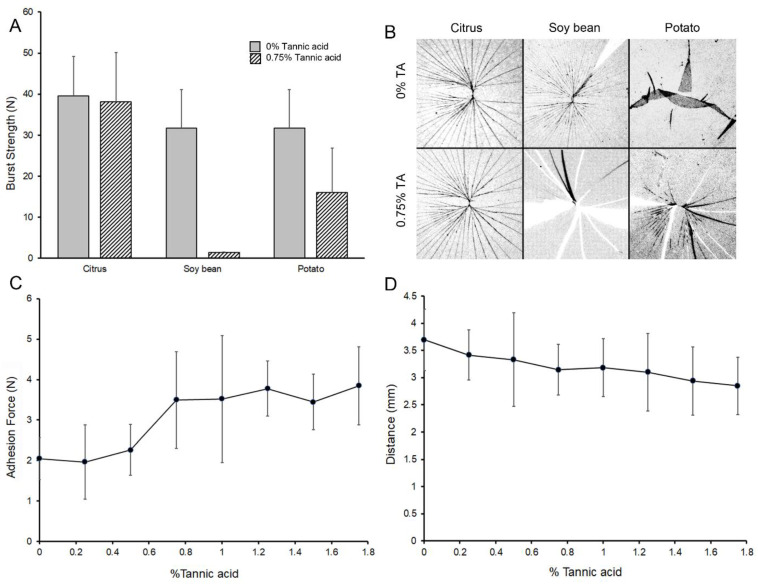
Effect of tannic acid on the physical properties of citrus pectin. Three pectin variants–citrus, soy bean and potato pectins–were analyzed for the effect of tannic acid on their physical properties in the glass phase. (**A**) Standard burst testing with a 5 mm stainless steel ball impacted the films at 2 mm/sec with simultaneous distance and force recordings. All films were studied at 5% water content (citrus 5.1 ± 0.8, soy bean 5.2 ± 0.2 and potato 5.4 ± 0.4). An intermediate concentration of tannic acid (0.75%) was chosen for detailed testing. Citrus pectin did not show a difference in burst strength with the addition of tannic acid. In contrast, soy bean and potato pectin films demonstrated significantly lower burst strength (*p* < 0.001). (**B**) Fracture patterns reflected the cohesion measurements. Citrus pectin showed little effect of the tannic acid; however, both soy bean and potato pectin demonstrated brittle films. (**C**) The adhesive force between citrus pectin films was increased with increasing concentrations of tannic acid (*p* < 0.05). (**D**) The extensibility of the pectin films was lower, but not statistically significant, with increasing tannic acid concentrations (*p* > 0.05). Error bars = 1 SD.

The effect of tannic acid on the physical properties varied with the source of pectin. Citrus high-methoxyl pectin, the pectin source used in the previous experiments, demonstrated no significant change in pectin film burst strength. In contrast, tannic acid weakened the burst strength of pectins derived from soy bean and potato sources (Figure 7A). Consistent with the burst strength findings, the fracture patterns of citrus pectin were similar with and without tannic acid. Soy bean and potato films demonstrated significant changes in fracture patterns with the addition of tannic acid (Figure 7B). Finally, tannic acid did not diminish pectin-pectin adhesivity (Figure 7C) nor did it significantly impact citrus pectin extensibility (Figure 7D).

## 4. Discussions

In this report, we developed a microfluidics system to quantitatively evaluate the facial erosion of pectin films. The system had 3 components: (1) a fluorescein tracer embedded in the pectin free volume, (2) a wettable porous matrix interface, and (3) a microfluidics perfusion system that facilitated multiple parallel comparisons. Signal isolation was enhanced with the incorporation of trypan blue as a quencher of released fluorescence. The utility of this system to evaluate facial erosion was demonstrated with the study of tannic acid. A potential pectin chain crosslinker, tannic acid demonstrated a highly significant dose-dependent modification of pectin erosion.

There are multiple definitions of biodegradability [19]. Here, the term biodegradability refers to the degradation of polymers subjected to a physiologic environment where the polymers are broken down into nontoxic fragments (monomers) and eliminated over time [20]. Although physiologic environments offer many potential biochemical reactions, we assume that the reaction with water is the dominant degradation process for pectin in vivo. Hydrogels are degraded by hydrolysis of the polymer backbone and/or hydrolysis of the cross-links [21]. A common approach to testing the rate of biopolymer hydrolysis is water immersion. Immersion testing simply involves immersion of the film in water followed by quantifying the polymer mass loss as a function of time. Although the testing is straightforward, the rapid rate of physical dissolution (minutes) complicates attempts to evaluate the primary physical process or the effectiveness of biodurability modifiers. Moreover, the rapid rate of dissolution observed in immersion testing is inconsistent with biological observations. In contrast to the rapid degradation in immersion testing, the estimated half-life of pectin films is 7 days in vivo [12].

To provide a more quantitative measure of biodegradation as well as a more physiologic microenvironment, we developed a microfluidics assay designed to explore the facial erosion of pectin films. To facilitate wetting and avoid flow artifacts at the pectin surface, we screened a variety of porous matrices to create the facial interface. Most of the screened membrane filters were composed of cellulose nitrate, cellulose acetate, or less refined pulp filters. The optimal synthetic porous membrane had a pore size that optimized lateral diffusion while minimizing penetrating flow artifact.

Critical to the highly quantitative measure of facial erosion was the use of fluorescein tracers embedded in the pectin films. The use of fluorescein tracers reflected the presence of a free volume within the pectin polymer. The free volume represents the unoccupied volume of the pectin film. Since this volume cannot be measured and theoretical models are uncertain [22,23], we empirically embedded fluorescein into the pectin film. Informed by experience using pectin as a drug delivery vehicle [1], we tested the physical properties of the tracer-embedded pectin films. The fluorescein tracer provided a strong fluorescence signal with no discernable effect on the pectin films’ physical properties. The pectin films with embedded fluorescein tracer demonstrated nearly identical physical properties to pectin films without the tracer. Notably, the burst strength, an objective measure of the films’ cohesive properties, was unchanged with the addition of the fluorescein tracer.

To improve signal isolation of the embedded tracer, we used trypan blue to quench the released fluorescein. A textile dye and vital stain [24], trypan blue is an effective fluorescence quencher [25,26]. Trypan blue had no measurable biochemical effect on the pectin polymer. At a standard 0.4% *w*/*v* concentration, stock trypan blue solutions provided both fluorescence quenching and water erosion.

The advantage of protracted degradation is the opportunity to quantitatively evaluate modifiers of pectin biodurability. An effective modifier was tannic acid. Tannic acid is a polyphenolic compound with multiple potential interactions with the heteropolysaccharide pectin including hydrogen bonding, hydrophobic interactions, covalent bonding, and electrostatic interactions. The most likely interaction, however, is hydrogen bonding. Tannic acid’s abundant phenolic groups serve as a hydrogen donor to link with hydrogen acceptors to form a stronger hydrogen bond [27]. In this case, tannic acid may function to increase chain interactions and decrease solubility by substituting for the water-pectin chain interactions. The size of the tannic acid molecule also suggests the potential for crosslinking between polymer chains. Regardless of the mechanism, the results of our microfluidics system suggest that tannic acid will have a significant impact on pectin durability in vivo.

Finally, the release of the embedded fluorescent tracer has obvious implications for drug delivery. Pectin is an extraordinary bioadhesive with the ability to adhere to the surface of virtually all visceral organs [9]. In addition to targeting visceral organs, the data here indicate that the pectin matrix can effectively deliver substantial concentrations of embedded drugs. The ultimate in situ biodegradation of the pectin is an additional attractive feature of pectin embedded drug delivery.

## Data Availability

The data presented in this study are available on request from the corresponding author.

## Abbreviations

PPS: points per second; RFU: relative fluorescence units; SD: standard deviation; TA: tannic acid; TB: trypan blue.
